# Lessons learned from two multicentre randomised controlled trials undertaken in pharmacies in community settings: a retrospective project management analysis

**DOI:** 10.1186/s13063-025-09196-9

**Published:** 2025-10-31

**Authors:** Christopher J. Byrne, Sarah K. Inglis, Andrew Radley, Lewis J. Beer, Minh D. Pham, Kate Allardice, Nicki Palmer, Brendan Healy, Joseph S. Doyle, John F. Dillon

**Affiliations:** 1 Division of Respiratory Medicine and Gastroenterology, University of Dundee, Ninewells Hospital and Medical School, Dundee, Scotland; 2 Directorate of Public Health, Kings Cross Hospital, NHS Tayside, Dundee, Scotland; 3 Tayside Clinical Trials Unit, University of Dundee, Ninewells Hospital and Medical School, Dundee, Scotland; 4 School of Health Sciences, City Campus, University of Dundee, Dundee, Scotland; 5 Department of Epidemiology and Preventive Medicine, Monash University, Melbourne, Australia; 6 Disease Elimination Program, Burnet Institute, Melbourne, Australia; 7 Department of Microbiology and Infectious Diseases, Public Health Wales, Cardiff, UK; 8 Department of Infectious Diseases, The Alfred and Monash University, Melbourne, Australia; 9 Department of Gastroenterology, NHS Tayside, Ninewells Hospital and Medical School, Dundee, UK

**Keywords:** Randomised controlled trials, Lessons learned, Project management, Community pharmacy

## Abstract

**Background:**

Community pharmacists are the most publicly accessible health professionals in high-income countries. There is increasing interest in conducting randomised controlled trials (RCT)—the benchmark of original evidence in the medical field—in community pharmacies. However, little evidence exists examining the challenges and opportunities of conducting RCTs in pharmacies, particularly with respect to project management. In this work, we aim to provide a narrative of lessons learned in conducting two RCTs in community pharmacies in two high-income countries.

**Methods:**

We retrospectively reviewed multiple data sources including administrative and trial activity records. We conducted face-to-face and online sessions to create a list of lessons learned from our experiences and created a stakeholder map for the trials examined. We framed our findings using the Project Managements Institute’s model of the project life cycle, and descriptive statistics were used to estimate the outcomes reported.

**Results:**

Ninety-six pharmacies were recruited. Across the project phases, seven high-level tasks within Initiation; 30 within Planning, 43 within Execution/Monitoring and Controlling, and 14 in Closure, were identified. Recruitment of pharmacies, developing documentation for trial drug supply, participant recruitment, and negotiation with pharmacy contracting bodies took longest to complete. Eight key stakeholder groups were identified, including public services/agencies; community pharmacies, communications actors; funders/sponsors; universities/research institutes; healthcare providers; suppliers; and regulators. Thirty lessons were identified, most critically across engaging and managing stakeholders; selecting and supporting trial sites; streamlining trial processes to minimise burden; optimising data collection and accuracy; and considering pharmacy constraints and costs.

**Conclusions:**

Conducting RCTs in community pharmacies presents unique challenges across the project life cycle. Key lessons include the importance of early planning, streamlining processes to reduce staff burden, and addressing pharmacy constraints. The complexity of the work necessitates dedicated and well-resourced trial management staff to enhance stakeholder management and offer tailored supports where required.

Trial registration.

Not applicable.

**Supplementary Information:**

The online version contains supplementary material available at 10.1186/s13063-025-09196-9.

## Background

Multicentre randomised controlled trials (RCT) are the benchmark of original evidence in the medical field and form the bedrock of translating hypothesised improvements in care into standard clinical practice [1, 2]. Although fundamental to decisions that are taken about healthcare delivery, there are myriad challenges to conducting RCTs including, but not limited to, selecting appropriate outcomes; defining eligibility criteria; identification and recruitment of trial sites to enrol adequate quantities of patients in an ethical manner; provision and adequacy of trial staff training; recruitment to target and retention of participants through the trial; and ensuring minimal participation burden upon trial participants [3–11]. As a result, many trials fail to fully recruit, collect spurious or inadequate data, incur substantial costs in re-recruiting trial sites and staff, and occasionally, may not publish results [7, 12–14].

RCTs often require, in addition to chief investigators, well-resourced cross-functional teams whose purpose is to facilitate delivery of the work. Dedicated trial management teams, including managers, coordinators, and assistants; data managers and clerks; and corollary administrative team members, or trial-specific staff (e.g. health economists), form inter-related elements of such teams [15]. Subsequently, the delivery processes of an RCT may be viewed through the prism of project management (PM) frameworks. Indeed, the United Kingdom (UK) Trial Managers Network frame the mechanisms required to meet trial objectives in unambiguously PM-grounded language: adherence to scope/quality; budget management; realistic and re-visited timelines; risk mitigation; efficient scheduling; resource monitoring; and information sharing [16]. Others suggest the trial manager—recommended for all primary research projects—should have strategic, tactical, and operational skills to plan and execute the project [15]. *Actively* managing *every* aspect of a given RCT has been suggested as the critical facilitator of trial success [16]. However, RCTs are rarely conducted within the confines of a single organisation, and even within organisations (across functions) the ability of a trial manager to influence outcomes varies [17]. This makes active management of every aspect of an RCT extremely challenging, leading to slippage in one or more of the three pillars of the PM triple constraint: time, scope, and cost [18].

In this study, we retrospectively applied a PM framework to the conduct of two multicentre RCTs we undertook in community pharmacies in the UK and Australia. The RCTs were trials of pharmacy-based diagnosis and treatment of hepatitis C virus (HCV) for people receiving routine opioid agonist therapy (OAT) for illicit drug dependence [19, 20]. These trials formed part of a wider suite of research examining novel approaches to HCV care in people who use drugs (PWUD) [21–29]. These approaches included trialling diagnosis and treatment for HCV in multiple novel community settings (e.g. pharmacies, needle and syringe provision sites, prisons, drug treatment centres) to generate evidence to enable progress towards elimination of HCV as a public health threat among PWUD.

Community pharmacists are the health professionals most accessible to the public, providing services at times often considered out of hours by other health service areas. In the countries the trials examined here were undertaken, governments have been keen to leverage the skill sets of pharmacists in community settings to provide a wider range of services in addition to their conventional roles [30, 31]. As part of this, community pharmacists are being encouraged to become involved in healthcare research, to provide evidence for the best ways to expand their services [32]. The Royal Pharmaceutical Society in the UK, and the Government in Australia, actively encourages pharmacists to become involved in research and has produced resources aimed at facilitating clinical research in pharmacies in community settings [33, 34]. To date, however, little literature exists documenting the challenges and opportunities of conducting RCTs through community pharmacies from a trial management perspective, despite the increasing popularity of these venues for intervention delivery across the continuum of human health.

We therefore sought to address the following questions in our work:

1. In the context of undertaking RCTs within community pharmacies, which actions can be identified within each trial management process outlined in our unit’s clinical trial route map [35], who actioned these, and to which domains of the Project Management Institute’s (PMI) project lifecycle can they be allocated?
2. How long did it take to reach key milestones from initiating antecedent process(es)?
3. Who are the stakeholders involved in delivering RCTs in community pharmacies in UK and Australia?
4. What lessons can be identified within each project phase for others seeking to conduct RCTs in community pharmacies?

Through these questions, we aim to provide a useful commentary of lessons learned, aligned to the PMI’s phases of a project, for other researchers seeking to undertake RCTs in community pharmacies in the UK and Australia.

## Methods

### Overview of the trials

Trial protocols and results are published elsewhere [19, 20, 23, 29]. One was conducted in the UK only (hereafter ‘SuperDOT-C’, NCT02706223) and one had UK and Australian sites (hereafter ‘Reach’, NCT03935906) [36, 37]. Each trial followed a cluster-randomised design; pharmacies functioned as the unit of randomisation and were randomised 1:1 to conventional care or the intervention arm. All OAT clients at a given pharmacy therefore received conventional care or the intervention. Pharmacists (SuperDOT-C) and/or nurses (Reach) delivered diagnosis of HCV and prescription of treatment within pharmacies. Treatment was dispensed by pharmacists alongside routine OAT provision for pharmacy clients. No incentives were provided. The trials were purposefully designed to minimise disruption to participants by providing all trial activities in an environment they routinely attended in their day-to-day life.

### Trial management

Both trials received trial management resources through Tayside Clinical Trials Unit (TCTU), a full service UK Clinical Research Collaboration-registered trials unit in Scotland [38].

### Study design

Similar to previous work, we used the PMI's model of the life cycle of a process-driven project, comprised of five high-level phases—Initiating, Planning, Executing, Monitoring and Controlling, and Closing—to frame our findings [39, 40]. However, as they occur simultaneously, we combined Executing with Monitoring and Controlling, resulting in four phases. Initially, the trial managers and coordinators retrospectively reviewed multiple data sources including administrative (grant applications, funding documents, study trackers, meeting agendas and minutes, email communications, data entry documentation) and conduct (approvals, trial activities, close-out) records to generate our time estimates. Time was measured in days from the start of a given task(s) to its terminating event (e.g. approval to first patient first visit).

Concordance between PMI project phases and the TCTU route map, and allocation of trial activities to PMI phases, was undertaken by CJB (PMI Certified Associate in Project Management, registration 8500944), SKI (Senior Trial Manager), and LJB (Trial Manager), and categorised by consensus [35]. The same individuals abstracted detailed activities into high-level tasks (a segment of work defined by the outcome desired, rather than the detailed administrative steps to get there) to aid interpretation. Collaborative stakeholder analysis was undertaken by all authors (Chief/Principal Investigators, trial management and coordination staff, and trial nurses) using MindView 8, a mapping software package. [41].

Finally, multiple face-to-face group sessions with Chief/Principal Investigators were conducted to create a list of lessons learned in light of our experiences conducting these trials; results of these sessions were shared remotely with all authors (which included Chief/Principal Investigators, trial management and coordination staff, and trial nurses) for input and further development.

### Ethics approval and consent to participate

Not applicable: this work did not require human or animal ethical approval.

### Statistics

Descriptive statistics were undertaken to derive counts, proportions, and averages, using Microsoft Excel 365.

## Results

Across the two trials, 96 community pharmacies were recruited as sites, all of which had at least one pharmacist trained in the relevant study protocol. Within those pharmacies, 600 participants consented to take part.

**Fig. 1:**
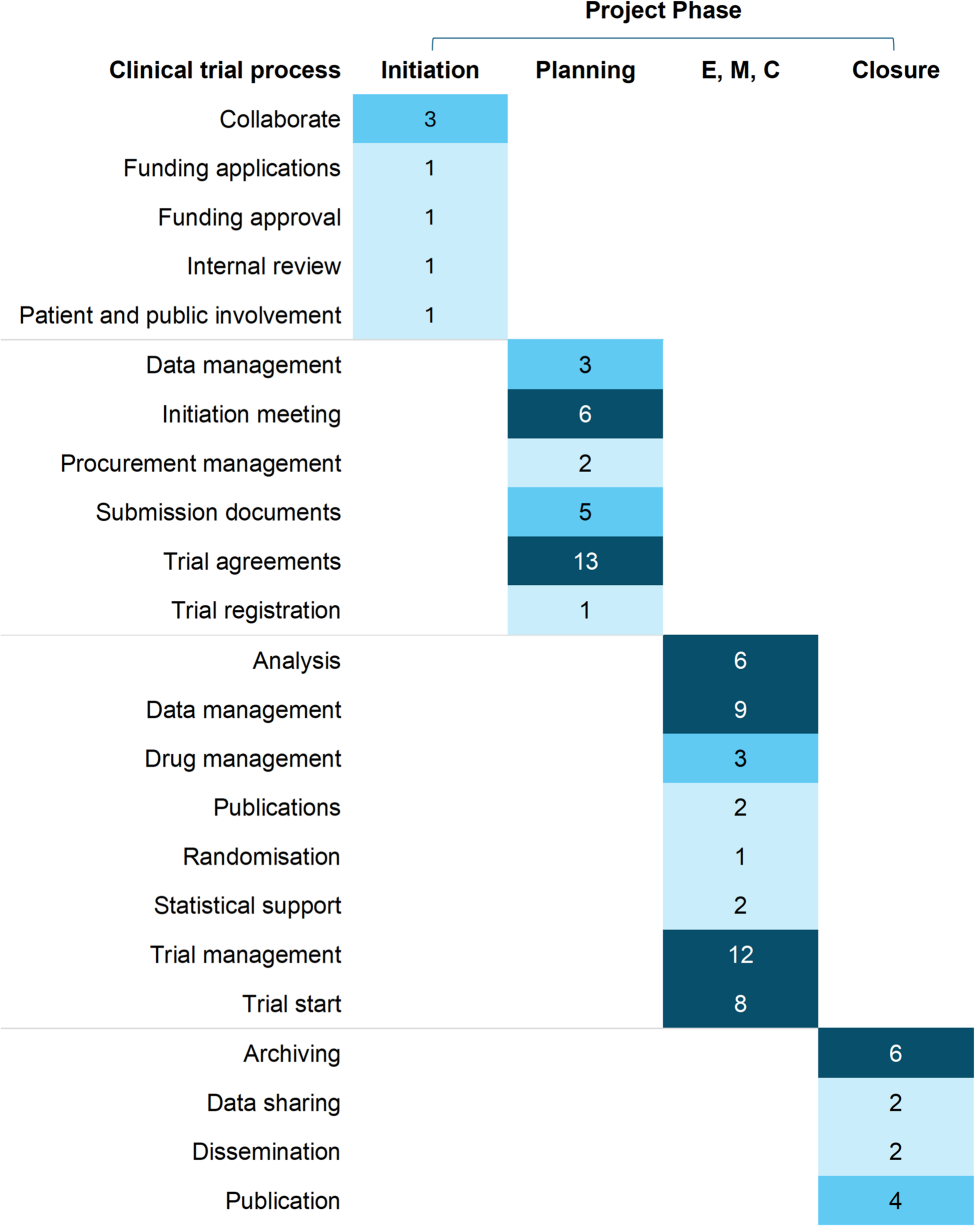
Number of high-level tasks linked to clinical trials route map processes across the project phases, providing a summary view of the route map processes with the highest density of high-level tasks. Note: Darker shading denotes a higher number of tasks relative to lighter shading. Processes are sorted alphabetically for each project phase. Abbreviations: E, M, C, Execution, Monitoring, and Controlling; Aus, Australia; NHS, National Health Service

TCTU facilitated delivery of both of these trials, and we identified seven high-level tasks within initiation; 30 within planning, 43 within execution/monitoring and controlling, and 14 in closure that we could link to processes specified in TCTU’s clinical trial route map (Fig. 1 and Table S1 [Supplementary File 1]). Most were led by trial management and coordination staff, followed by investigators and analytical staff (Figure S1 [Supplementary File 1]).

Although most actions were relevant to all trial settings, five were considered to be specific to trials conducted in community pharmacies. These all fell within the Planning phases, and concerned securing legal agreements with pharmacy contracting boards, pharmacy chains, individual/independent pharmacies, as well as developing and implementing patient group directions and service specifications (Table S1, Supplementary File 1).

In the analysis of key deliverables, we found recruitment of pharmacy sites and the development of key documents for trial drug administration (patient group directions [PGD]) took the longest time in the early phases of the trials (Table 1).

**Table 1:** Time to completion of key trial deliverables

	**Process**	**Days – Med (IQR)**
Setup	Sponsor: submission to approved	42 (23)
	Research ethics: submission to approved	49 (24)
	R&D: first submission to all approved	48 (47)
	Sponsor submission to CT.gov published	99 (57)
	Pharmacy contractor negotiations	63 (51)
	Recruitment of pharmacy sites	210 (198)
	Patient group directions: draft to live versions	124 (113)
Progress	Protocol amendments: submission to implementation	17 (39)
	FPFV to LPLV	391 (122)
	First patient consent to final patient consent^a^	133 (227)
	Database development to live	183 (34)
	Data entry start to data locked	354 (52)
Post	Data lock to publication	427 (49)
	Data lock to archive	883 (442)

*Med* median, *IQR* interquartile range, *R&D* research and development, CT.gov, www.clinicaltrials.gov, *FPFV* first participant first visit, *LPLV* last participant last visit

^a^Per pharmacy that consented at least one participant

As the trials progressed, recruitment and completion of trial visits, database development, and data entry, unsurprisingly, had the highest average time associated with them, as well as eventual archiving of trial materials. Negotiation with pharmacy contracting committees (a key process to secure access to pharmacies as research sites) took us, on average, close to 3 months to conclude successfully, while the completion of the open trial registrations on our chosen platform (clinicaltrials.gov [CT.gov]) was found to be time consuming, perhaps reflecting the time it can take for CT.gov to review and release study records. However, review and receipt of approvals, including for amendments to protocols, were found to be promptly completed.

**Fig. 2:**
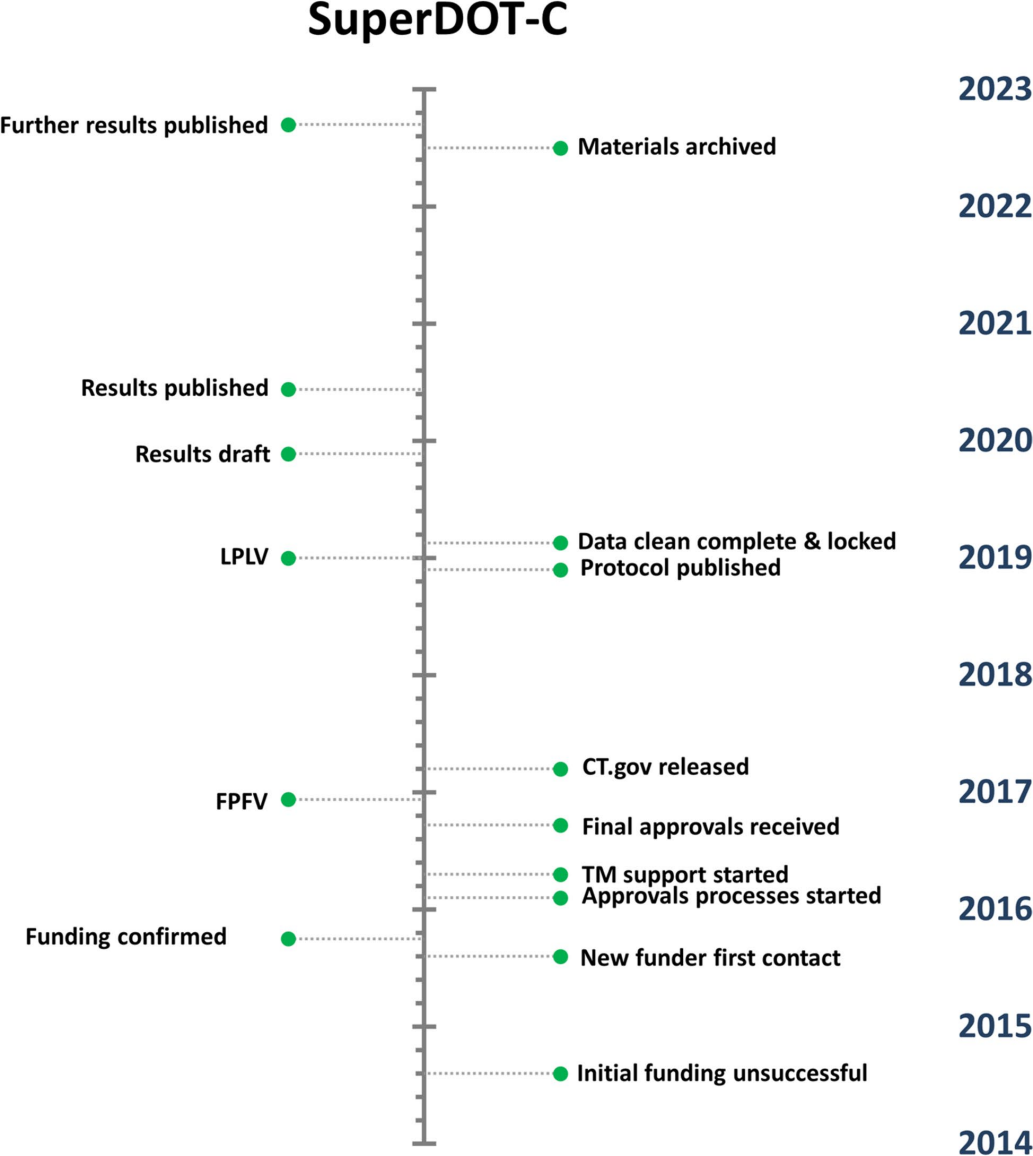
Timeline with key milestones for the SuperDOT-C trial. Abbreviations: TM, trial management; FPFV, first patient first visit; CT.gov, www.clinicaltrials.gov; LPLV, last patient last visit

**Fig. 3:**
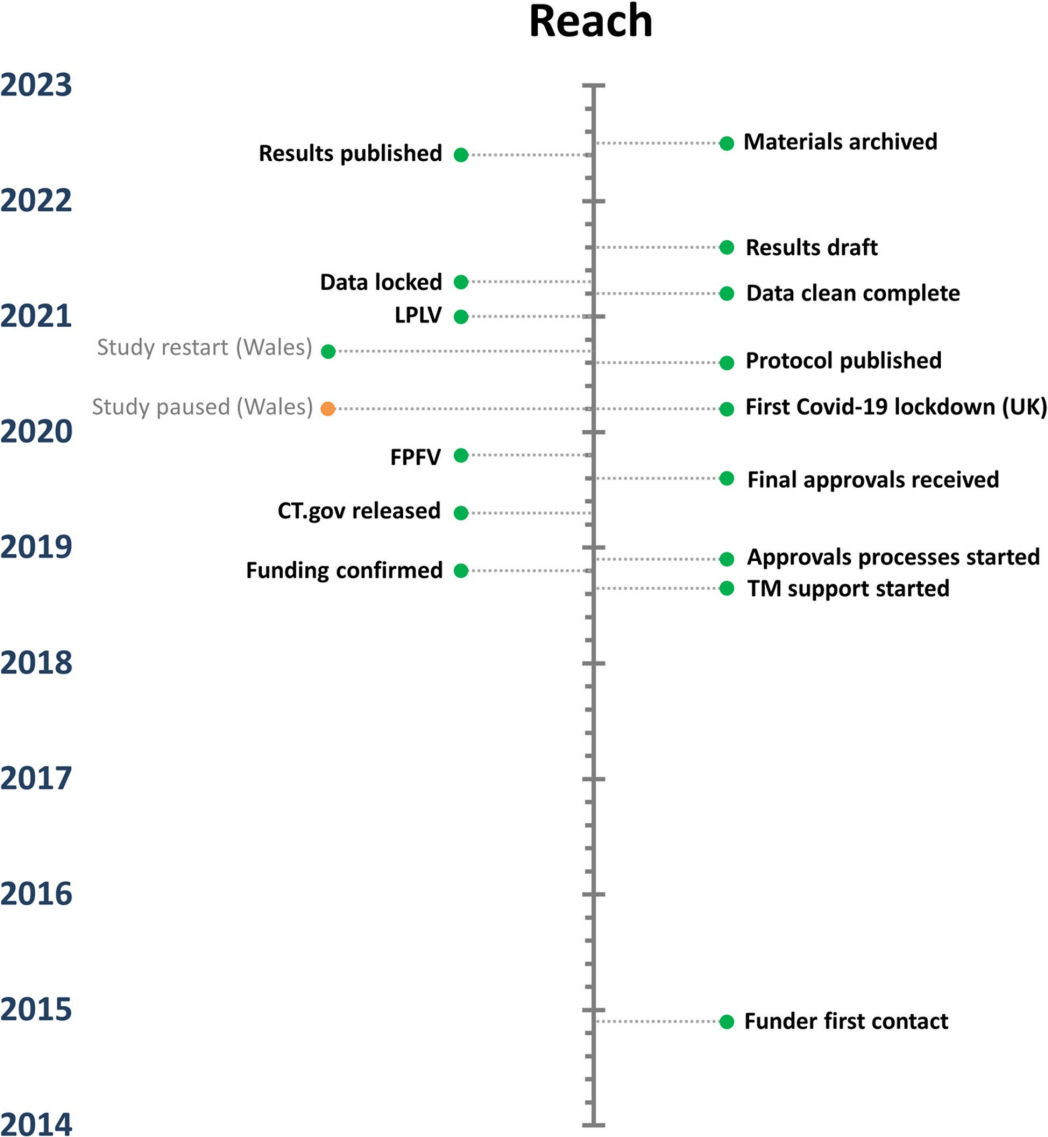
Timeline with key milestones for the Reach trial. Abbreviations: TM, trial management, CT.gov, www.clinicaltrials.gov, FPFV, first patient first visit, UK, United Kingdom of Norther Ireland and Great Britain, LPLV, last patient last visit

The overall timeframes involved in delivering trials in community pharmacies, from funding applications to archiving of materials, were found to span multiple years (Figs. 2 and 3). This reflects the complexity of project funding, planning, delivery, and ultimate conclusion.

The funding process was substantially quicker to complete for SuperDOT-C (Fig. 2) than it was for Reach (Fig. 3), reflecting the challenging nature of securing funding for trials in community pharmacies. SuperDOT-C was publicly funded, with support in kind from pharmaceutical companies and the National Health Service (NHS), while Reach was wholly funded by a pharmaceutical company; it may be that public funders are more receptive to supporting trials conducted in this setting, than private funders. That said, SuperDOT-C was proposed to multiple funders, whereas Reach only went to one for consideration. Also noted was that the archiving process took substantially longer for SuperDOT-C than for Reach; this was impacted substantially by work-from-home mandates necessitated by the COVID-19 pandemic.

**Fig. 4:**
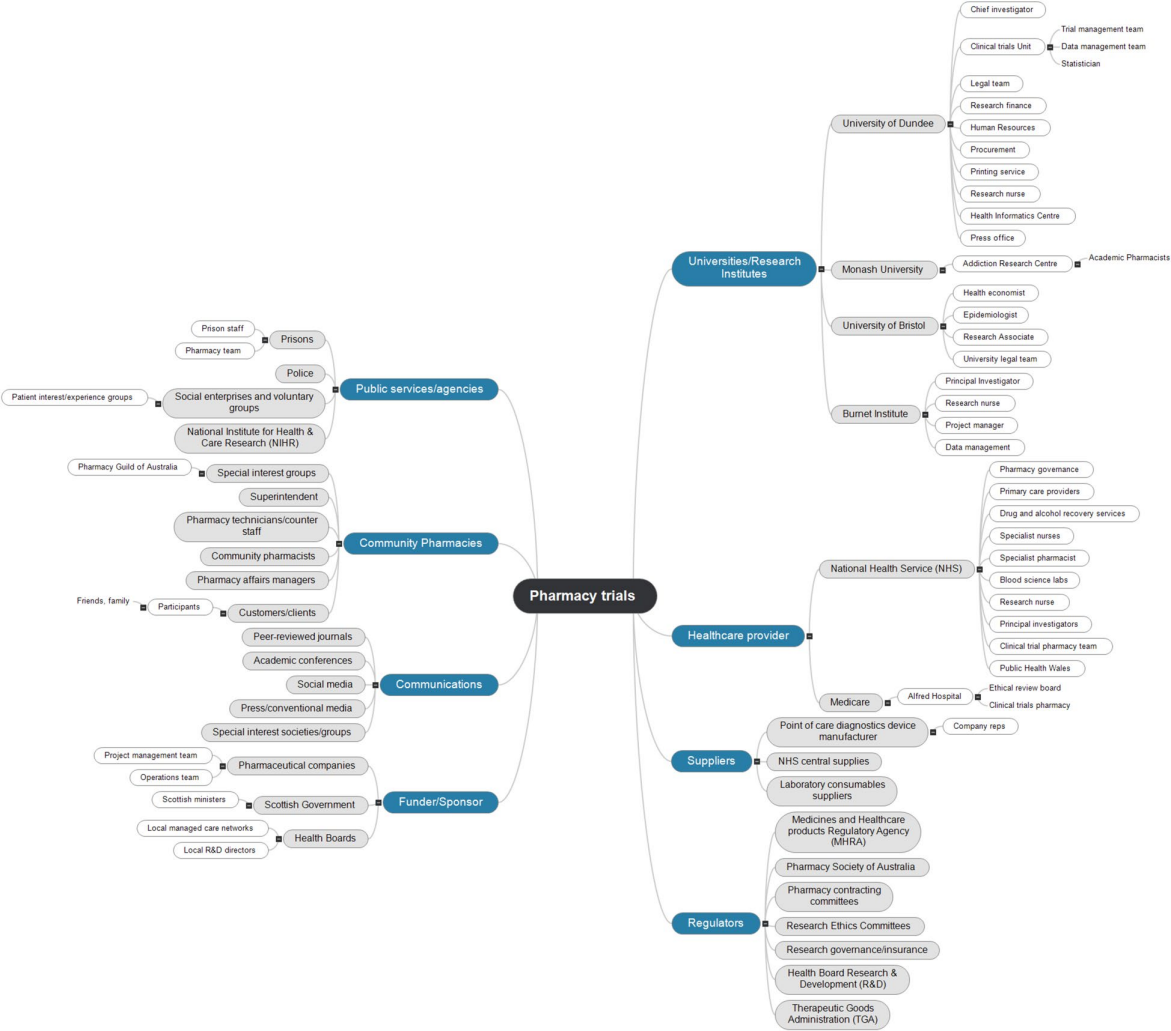
Results of stakeholder analysis showing key actors involved in planning and delivery of clinical trials in community pharmacies. Abbreviations: R&D, research and development

In the stakeholder analysis, we identified eight key groups of stakeholders who were involved in trial activities. These included public services/agencies; community pharmacies, communications actors; funders/sponsors; universities/research institutes; healthcare providers; suppliers; and regulators (Fig. 4).

These were extended further to identify 34 sub-groups that were important to the delivery of these trials. These stakeholders cut across agencies, institutions, and individuals, embedded in both the public and private sectors, and serve to illustrate that while a core team is responsible for planning and delivering trials in these settings, many indirect stakeholders can influence the ultimate successful (or not) completion of trials in pharmacies. This underscores the importance of undertaking a stakeholder analysis early in the planning of these trials, and regularly revisiting and updating this to ensure those with roles and responsibilities which can impact upon outcomes are engaged appropriately by core trial teams.

When considering the reported data in its totality as part of in-person discussions, and following remote stakeholder review and input, we identified 30 lessons learned across the four PMI phases of a project which we believed to be beneficial to disseminate. These are presented here according to each phase.

### Initiation

Within the initiation phase, we identified eight lessons learned. These cut across funding (already mentioned), trial design, patient and/or public involvement, target sites for recruitment to the trial, the heterogeneity of the physical environment in pharmacies, and liaison with critical actors for input into your trial in the early stages.

1. Study funders may take inordinately long to review trials based in pharmacies, as they are not very common. Therefore, we suggest engaging with potential funders early and often to develop the research proposal.
2. Trials in pharmacies can be challenging to design, particularly with planning sample size and statistical approaches (differences in intervention uptake are typically large between sites). Engage with researchers/trials units with experience of trials in community pharmacies early for advice and input on protocol design.
3. There was little evidence to suggest trialling the interventions we evaluated in pharmacies would be attractive to the patients involved, and early pilot work changed multiple aspects of our trial designs. Therefore, explore opportunities for patient/public input to your proposal. Where this is unavailable, seek advice from charity and clinical services involved in service delivery for your target population and explore opportunities for pilot work.
4. Try to establish which pharmacies in your recruiting region are most frequented by the patient population you seek to recruit. This may be possible by engaging local/regional public health directorates to assist with accessing relevant anonymised data, or national pharmacy societies.
5. The quantity and quality of consulting spaces to action trial activities within pharmacies varies substantially. While you shortlist pharmacies you are interested in, make a list of site criteria to check candidate pharmacies against before contracting (e.g. adequate private space, access to electricity, clinical waste, sanitation).
6. The way(s) to approach pharmacies to recruit to trials can be unclear and heterogenous. For example, independent smaller pharmacies may be contacted directly, while larger chains may have large research governance structures. Explore possible mechanisms for recruiting community pharmacies, or pharmacy chains, long before planning your trial as it is time consuming. Make use of any contacts within local health services to facilitate this, but if this is not possible then make contact in the first instance with local/regional pharmacy managers or relevant commissioning boards, professional guilds or associations.
7. Document potential constraints of undertaking the trial within community pharmacies and develop the study protocol to account for them (e.g. adverse event monitoring).
8. Liaise with relevant community pharmacy bodies (e.g. Community Pharmacy Scotland/Wales; Pharmacy Guild of Australia) early on about developing service specifications, approval processes, timeframes, etc., and what costs are attached to possible interventions. Some costs may be paid for by excess treatment costs (UK only) but if not, these would need to be passed on to the funder.

### Planning

Within the planning phase we identified eight lessons learned. These cut across planning for who to involve in supporting your trial, securing agreement from sites/pharmacies to participate in the trial, medication supply, our experience of patient dynamics, the supports required for community pharmacists from secondary care, and training and turnover rates for pharmacy staff.

1. Liaise with community pharmacy bodies, health boards/trusts, to identify key players within community care and pharmacy commissioning teams, who can support your work. Consider inviting these individuals to consult on trial implementation/documentation for sites.
2. There is an opportunity cost for pharmacies participating in research. Independent pharmacies typically agree participation terms quickly, while large-chain pharmacies tend to take longer. Therefore, begin contract negotiations, particularly with large chains, as soon as possible, while negotiations with independent pharmacies can follow.
3. If using non-market authorised medication for your trial, liaise closely with your partner clinical trials pharmacy to develop dispensing processes for any Investigational Medicinal Product (IMP) which may need to be provided to community pharmacies, as secondary dispensing concerns may mean that the local pharmacy team are unable to take responsibility for issuing a medicine. Safe guards should be designed.
4. Perhaps more often than in hospital-based or inpatient trials, participants of community trials experience sudden and unexpected changes in circumstances which may impact their participation (e.g. living arrangements). Create mechanisms for interaction between community pharmacies and local statutory, health, and social services, so that affected individuals are not disadvantaged where possible.
5. Community pharmacists sometimes require support from tertiary specialists in the disease under investigation. Establish good communication pathways to specialist advice, e.g. set up encrypted email addresses for pharmacists/pharmacies, and enable access to online laboratory test results and other relevant systems, in tertiary care.
6. If you will seek pharmacists to prescribe/supply trial medication, be mindful that not all of them will be qualified independent prescribers. Explore whether a PGD can be established for your trial medication, if using one, to simplify workflow(s) and enable you to recruit from a wider pool of pharmacies.
7. Pharmacy staff will likely be inexperienced in undertaking research and not have typical qualifications held by researchers, such as Good Clinical Practice. Provide bespoke training, including materials that can be used remotely by new staff joining the study after site initiation.
8. Community pharmacies have high staff turnover rates in our experience, so train as many people as possible at each site, including pharmacists, pharmacy technicians, and counter staff, in trial activities. Budget for re-training.

### Execution, monitoring and controlling

Within the execution, monitoring, and controlling phase we identified ten lessons learned. The topics spanned pharmacy staff training and support, monitoring and supply of trial consumables, communications and awareness raising within the health service, and data collection methods.

1. If possible, conduct trial training away from the live pharmacy environment. Delivering trial training in an open pharmacy is challenging (funding is required for this, to cover locum costs; this should be considered in the initiation phase) and interruptions are frequent.
2. Pharmacy staff will need regular support to comply with the study protocol. Employ dedicated study coordinators ensuring a single point of contact for community pharmacy staff requiring assistance, and to give pharmacy staff a familiar face thereby enabling regular light-touch monitoring of site activities.
3. Pharmacies are not typically large enough to hold substantial amounts of trial consumables on premises, or may not have specific equipment required for your trial. Ensure coordinators pro-actively monitor site stock levels—pharmacists are busy and often reach out at the last minute—and resupply as required throughout the trial.
4. Notify local primary care providers about the study before opening to recruitment so they will have an awareness should any patients under their care participate.
5. There is a cost (time) and risk (non/inaccurate completion) to completing trial paperwork in community pharmacies. Make the burden as light as possible and anticipate substantial monitoring and assistance through trial staff.
6. Pharmacy environments are hectic. We used paper-based case report forms for data collection, adverse event (AE) reporting, and monitoring of dispensing, which quickly degraded at some sites. We would recommend implementing an electronic version of these documents if possible.
7. Where health data is protected by confidentiality regulations, ensure secure communications between pharmacy sites and the health service(s) supporting the trial are used (e.g. encrypted email) by study staff.
8. Trial activities may be less onerous on pharmacists where they have digital access to health records which are required for trial activities. Where possible, we suggest establishing access to these records for pharmacists.
9. Pharmacy staff are extremely time pressured. Subsequently, ensure the data being collected is the very minimum required to reduce the likelihood of missing or inaccurate data.
10. Plan for expected AEs in your study population and design reporting procedures around them for pharmacists, ensuring prompts to check and report AEs by pharmacy staff.

### Closure

Finally, in the closure phase, we identified four lessons learned. Principally, these focussed on disposing of trial medication, site closure, settling fees, and access to clinical systems.

1. Excess IMP can be destroyed on site at community pharmacies. Returns to clinical trials pharmacy are not usually required.
2. Depending on how you sequence your data queries, sites can be closed remotely by digital or phone communication. About 4 weeks’ notice is probably sufficient.
3. Liaise with relevant pharmacy research committees/contractors committees to pay any outstanding fees and confirm closure of pharmacy sites.
4. Ensure any access to clinical systems or NHS email systems is revoked for pharmacists if not continuing as a standard health service.

## Discussion

RCTs are complex projects which increasingly require specialist PM skills and experience to facilitate completion. In this study, reflecting on our experiences of delivery RCTs within community pharmacies, which remain unusual trial settings, we aimed to examine which actions are important to undertaking RCTs within these venues and align these to a recognised project management framework; we sought to understand the time commitment required to reach key milestones for such RCTs; we identified critical stakeholders to engage when delivering RCTs in pharmacies; and we generated a list of key lessons which may be useful to others seeking to deliver such trials. The findings illustrate that the time horizon for these RCTs can be up to 10 years and that the most time-consuming aspects are likely to be: securing funding; recruitment of pharmacy sites; administration to facilitate IMP supply; recruitment of participants; and data entry and archiving. We also found that the multitude of actions underpinning delivery of such trials across the phases of the project necessitate dedicated TM staff and resources to ensure success; funding applications should adjust for this. The necessity for this was illustrated by the many stakeholders involved in delivery which require active engagement and management to increase the likelihood of delivery to time and scope, and within budget.

Although there is not a substantial existing literature examining lessons learned during the conduct of RCTs, there has been increasing interest in this over time. Several publications have reported experiences across diverse areas such as: physiotherapy interventions in intensive care; agile management to setup research registries; development of remote digital interventions for cancer; and implementation of remote cardiorespiratory self-management models [40, 42–44]. One further study explored acceptability of different trial management methods with stakeholders involved in a trial of novel interventions for obsessive compulsive disorder [45]. In light of these works, our study is the first to our knowledge to report lessons learned for RCTS conducted in community pharmacies. Similarly to these studies, however, we found staff turnover to be an important challenge [40, 44]—which, in a similar fashion, necessitated multiple rounds of re-training at sites by trial staff—and establishing clear lines of communication and clinical support to be critical to success [43, 45]. Moreover, we also found that actively supporting sites with their trial paperwork with informal monitoring visits by trial staff assisted with accurate completion of CRFs, and other data collection paperwork, and timely reporting of AEs, which ultimately resulted in good fidelity to trial procedures and cleaner data for initial entry [40].

Existing work has suggested identification of required tasks, and estimating the time needed to complete these, are important challenges for triallists to overcome as part of trial planning prior to commencement [43]. We therefore believe this study makes a useful contribution in identifying these to assist those planning future trials in community pharmacies. Although many we identified could apply to trials across settings—similar to existing published work [45]—we reported five which were unique to pharmacies. All were within the planning domain and related to formal agreements which must be in place. These ranged from recruiting specific sites (both chain and independent pharmacies), to agreeing participation terms with pharmacy contracting boards; developing PGDs (if applicable); and developing service specifications (if applicable). The latter two were specific to the trial designs employed in our studies. However, the approaches required to secure sites is reflected in the time taken to complete negotiations with pharmacy contractor panels (63 days) and pharmacies themselves (210 days). These negotiations were frequently framed by one of the lessons learned in the Planning domain (opportunity cost to participation), and necessitated several rounds of negotiating finances for delivery of specific study activities (e.g. per patient consent, per intervention delivered). Triallists should be prepared to go into this level of detail when recruiting pharmacies and have sufficient funds budgeted to make participation attractive to pharmacies, which ultimately are commercial entities. This will require early engagement prior to funding applications.

The identification of tasks necessitates the clarification of stakeholders involved to ensure those tasks are completed. While we identified many stakeholders that broach both the private and public sectors, some were perhaps less common to triallists than others. Specifically: prisons and prison staff, police staff, social enterprises and voluntary groups, and drug and alcohol recovery services. These organisations were perhaps unique to our trials—wherein the participants recruited were PWUD—and we interacted with them extensively in the execution phases of the trials. However, their identification speaks to a broader point: when recruiting participants in community settings, be prepared with contingency plans for sudden and unexpected changes in circumstances. Through flexible (though reactive) approaches by our trial staff, we facilitated continued participation for our participants while they found themselves unexpectedly under the care of custodial services; this necessitated close liaison with police and prison staff, as well as voluntary support agencies and recovery services. Other triallists should be mindful of any potential (and likely) changes in circumstances which may be likely to occur in their trial population in the community and create contingency plans which are informed by stakeholder analyses. This will ensure minimal disadvantage to participants.

## Limitations

As the trial managers and investigators were involved in the collection and analysis of data for this study, a bias may have been introduced in reporting and interpreting the findings. We tried to minimise this by being transparent about the methods and materials used, and undertaking a collaborative process with staff of varying roles and responsibility, and across settings, to input on and interpret the findings. Furthermore, the available documents we used for analyses were not created for this purpose, therefore some of the dates collected to estimate time may have been inaccurate, due to variable quality in records keeping. Additionally, meeting minutes were of variable length, details and quality, which may have introduced inaccuracies, or led to some potential lessons being omitted. Notwithstanding these limitations, we believe our study has several strengths in its novelty, potential utility to inform future pharmacy-based trials, and relevance to the evolving context of trials increasingly conducted in community settings.

## Conclusion

Ultimately, while there are challenges to conducting healthcare research in community pharmacies, there are benefits, which include accessibility for participants, and the potential to engage disadvantaged communities, who are often under-represented in healthcare research but live with higher-than-average disease burdens. This is aligned to the key need identified by the National Institute for Health and Care Research strategy for research inclusion (the need to widen access and participation for greater diversity and inclusion to enable people from all backgrounds to participate in research) [46]. Expensive and logistically challenging visits to hospital clinics (often the location for healthcare research), and distrust of researchers, can be significant barriers to participation in RCTs [47]. Close to 90% of the UK population live within a 20-min walk of a community pharmacy, and staff there are trusted by their clients (often more than they trust their clinicians) [48–50]. Basing a study within community pharmacies therefore may help to remove these barriers, engage a more diverse group of participants, and therefore narrow inequalities in participation in healthcare research. Our studies enrolled people who used drugs who experience myriad health-related inequalities which can lead to exclusion from health research; basing the trials in environments familiar to, and easily accessed by, this population was the key step in ensuring they were able to participate in the research.

In sharing lessons learned and some key metrics observed in our work, we hope to facilitate others considering undertaking research in this setting.

## Supplementary Information

Supplementary Material 1.

## Data Availability

Prospective data was not collected for this work. It was predominantly underpinned by routinely collected administrative data, which includes identifiable information. Consequently, this cannot be made publicly available.

## Abbreviations

AE: Adverse event
GCP: Good Clinical Practice
HCV: Hepatitis C virus
IMP: Investigational Medicinal Product
NHS: National Health Service
OAT: Opioid agonist therapy
PWUD: People who use drugs
PGD: Patient group direction
PM: Project management
PMI: Project Management Institute
RCT: Randomised controlled trial
TCTU: Tayside Clinical Trials
UK: United Kingdom of Great Britain and Northern Ireland
