# Supplementary material for: Lessons learned from two multicentre randomised controlled trials undertaken in pharmacies in community settings: a retrospective project management analysis
Source: Trials. 2025 Oct 31;26:461. doi: 10.1186/s13063-025-09196-9 (PMC12577213; doi:10.1186/s13063-025-09196-9)
Supplement: Supplementary file 1 — Supplementary Material 1. [file 13063_2025_9196_MOESM1_ESM.docx]

**Supplementary File 1**

**Title**

Lessons Learned from two Multicentre Randomised Controlled Trials undertaken in Pharmacies in Community Settings: a retrospective project management analysis.

**Authors**

Christopher J Byrne^1 2^ Sarah K Inglis^3^ Andrew Radley^2 4^ Lewis J Beer^3^ Minh D Pham^5 6^ Kate Allardice^6^ Nicki Palmer^7^ Brendan Healy^7^ Joseph S Doyle^6 8^ John F Dillon^1 9^

^1^Division of Respiratory Medicine and Gastroenterology, University of Dundee, Ninewells Hospital and Medical School, Dundee, Scotland.

^2^Directorate of Public Health, Kings Cross Hospital, NHS Tayside, Dundee, Scotland.

^3^Tayside Clinical Trials Unit, University of Dundee, Ninewells Hospital and Medical School, Dundee, Scotland.

^4^ Department of Population Health and Genomics, University of Dundee, Ninewells Hospital and Medical School, Dundee, Scotland.

^5^ Department of Epidemiology and Preventive Medicine, Monash University, Melbourne, Australia

^6^ Disease Elimination Program, Burnet Institute, Melbourne, Australia.

^7^Department of Microbiology and Infectious Diseases Cardiff, Public Health Wales, UK

^8^Department of Infectious Diseases, the Alfred and Monash University, Melbourne, Australia.

^9^Department of Gastroenterology, NHS Tayside, Ninewells Hospital and Medical School, Dundee, UK.

**Figure S1:** Number of high-level tasks led by role across project phases.

**Note:** Darker shading denotes a higher number of tasks relative to lighter shading. Roles are sorted alphabetically.

**Abbreviations:** E, M, C, Execution, Monitoring, and Controlling; Aus, Australia; NHS, National Health Service.

Note that the actions listed in Table S1 are not exhaustive, and but are intended as a reference to illustrate key actions and stakeholders involved in delivery.

| **Table S1:** Key tasks identified for each trial route map process, their respective PMI project phase, and the role with key responsibility for delivering. | | | |
| --- | --- | --- | --- |
| **Route map process** | **Task** | **Phase** | **Mainly actioned by** |
| Collaborate | Research team contact unit. | Initiation | Unit co-director |
|  | Meet to provide advice on study design. | Initiation | Unit co-director |
|  | Budget drafted and agreed. | Initiation | Unit co-director |
| Internal review | Resource review and allocation. | Initiation | Unit co-director |
| PPI | Advise on PPI requirements.  For these trials, this was based on focus group work in pilot studies. | Initiation | Chief investigator |
| Funding applications | External process; support researchers with queries. | Initiation | Researchers |
| Funding approval | Receive and administer funding, including financial agreements. | Initiation | University research finance team  Unit legal team |
| Initiation meeting | Kick off meeting | Planning | Unit co-director  Senior trial manager |
|  | Create review and sign off | Planning | Unit co-director |
|  | Write protocol | Planning | Trial manager |
|  | Create patient facing documentation | Planning | Trial manager |
|  | Create template data collection forms | Planning | Trial manager |
|  | Create template communications | Planning | Trial manager |
| Submission documents | Sponsor review | Planning | Trial manager |
|  | REC/HREC review | Planning | Trial manager  Research nurse (Aus) |
|  | R&D review | Planning | Trial manager |
|  | TGA review | Planning | Post-doctoral researcher (Aus) |
|  | MHRA review | Planning | Sponsor's office |
| Trial agreements | Health boards | Planning | Unit legal team |
|  | Funding agreements | Planning | Unit legal team |
|  | IMP supply agreements | Planning | Unit legal team |
|  | Pharmacy contractors* | Planning | Unit legal team  Principal investigator |
|  | Pharmacy chains* | Planning | Unit legal team |
|  | Individual pharmacies* | Planning | Unit legal team |
|  | Sponsorship agreement | Planning | Unit legal team |
|  | Site-specific information agreement | Planning | Unit legal team |
|  | Non-commercial research agreements | Planning | Unit legal team |
|  | Data sharing agreement(s) | Planning | Unit legal team |
|  | Site R&D agreements | Planning | Unit legal team |
|  | Patient Group Direction* | Planning | Principal investigator |
|  | Service specification* | Planning | Principal investigator  Trial manager |
| Trial registration | Register trial on public registry | Planning | Trial manager |
| Procurement management | Non-competitive action form | Planning | Trial manager |
|  | Sourcing suppliers | Planning | Trial coordinator |
| Data management | Create data management plan | Planning | Trial manager  Trial coordinator |
|  | Start database development | Planning | Trial manager  Trial coordinator |
|  | Create Case Report Form | Planning | Trial manager  Trial coordinator |
| Randomisation | Randomisation of pharmacies | E, M & C | Trial coordinator |
| Publications | Draft, revise, and submit protocol manuscript | E, M & C | Principal investigator  Trial manager  Statistician |
|  | Draft, revise, and submit protocol conference abstracts | E, M & C | Principal investigator  Trial manager  Statistician |
| Trial start | Site training - Protocol / GCP | E, M & C | Principal investigator  Trial manager  Trial coordinator |
|  | Nurse training (point of care device) | E, M & C | Manufacturer  Trial coordinator |
|  | Site initiation - community pharmacies | E, M & C | Trial coordinator |
|  | Site initiation - clinical trials pharmacies | E, M & C | Trial manager  Trial coordinator |
|  | Site initiation - conventional care sites | E, M & C | Trial manager  Trial coordinator |
|  | Trial staff delegation logs | E, M & C | Chief investigator  Principal investigator  Trial coordinator |
|  | Trial medication supply | E, M & C | Clinical trials pharmacist  Trial manager  Trial coordinator |
|  | Materials/consumables supply to pharmacies | E, M & C | Trial coordinator |
| Trial management | Participant recruitment | E, M & C | Community pharmacists  Trained pharmacy staff |
|  | Recruitment monitoring | E, M & C | Trial coordinator |
|  | Bloodwork result provision | E, M & C | NHS labs  Community pharmacists  Trial coordinator |
|  | Prescribing | E, M & C | Chief investigator  Principal investigator  Community pharmacists |
|  | Site communication | E, M & C | Trial coordinator  Trial nurses |
|  | Ad-hoc training (new staff) | E, M & C | Principal investigator  Trial manager  Trial coordinator |
|  | Materials/consumables re-supply to pharmacies | E, M & C | Trial coordinator |
|  | Administration of protocol amendments | E, M & C | Trial coordinator  Post-doctoral researcher (Aus) |
|  | Reporting | E, M & C | Trial manager  Trial coordinator |
|  | Payments to pharmacies | E, M & C | Trial coordinator |
|  | Participant retention | E, M & C | Trial Coordinator  Community pharmacists  Trained pharmacy staff |
|  | Site closures | E, M & C | Trial manager  Trial coordinator  Community pharmacists  Trial nurses  University research finance team |
| Drug management | Data monitoring committee | E, M & C | Chief investigator  Trial manager  Trial statistician |
|  | Monitor expiry dates | E, M & C | Clinical trials pharmacy technician  Community pharmacists  Trained pharmacy staff |
|  | Re-stock medication | E, M & C | Trial coordinator  Clinical trials pharmacy technician |
| Data management | Create data management plan | E, M & C | Trial manager  Trial coordinator |
|  | Scope data management system & CRF | E, M & C | Chief investigator  Principle investigator  Trial manager  Trial coordinator  Informatics centre |
|  | Print and distribute CRFs to sites | E, M & C | Trial coordinator |
|  | Complete CRFs | E, M & C | Community pharmacists  Trial nurses |
|  | Data cleaning | E, M & C | Trial coordinator |
|  | Data entry | E, M & C | Trial coordinator  Trial nurses |
|  | Data verification | E, M & C | Trial coordinator  Trial nurses |
|  | Data transfer | E, M & C | Informatics centre  Trial manager  Unit legal team  Trial coordinator |
|  | Database lock | E, M & C | Chief investigator  Statistician  Trial manager  Trial coordinator |
| Statistical support | Create statistical analysis plan | E, M & C | Chief investigator  Statistician  Trial manager |
|  | Create health economic analysis plan | E, M & C | Chief investigator  Statistician  Health economist  Trial manager |
| Analysis | Analyse data | E, M & C | Statistician  Health economist  Research associate |
|  | Provide table and figures | E, M & C | Statistician  Health economist  Research associate |
|  | Draft, revise, and submit manuscript | E, M & C | Chief Investigator  Trial manager |
|  | Draft, revise, and submit abstracts for conferences | E, M & C | Chief investigator  Trial manager |
|  | Draft, revise, and circulate press release | E, M & C | Trial coordinator  University press officer |
|  | Draft and circulate results newsletter to sites | E, M & C | Trial coordinator |
| Publication | Finalise and submit manuscripts and abstracts | Closure | Chief investigator  Statisticians  Trial manager  Trial coordinator  Funder |
|  | Share publications on social media | Closure | Trial coordinator |
|  | End of trial declaration to governance/REC/funders | Closure | Trial manager  Trial coordinator |
|  | Final report to funder | Closure | Trial manager  Trial coordinator |
| Dissemination | Newsletter | Closure | Trial coordinator |
|  | Press release | Closure | Trial manager  Trial coordinator  University press officer |
| Data sharing | Data sharing statement | Closure | Trial manager |
|  | Interact with interested parties | Closure | Trial manager |
| Archiving | Archiving request | Closure | Trial coordinator |
|  | Collate materials for archiving | Closure | Trial coordinator |
|  | Securely store identifiable data | Closure | Trial coordinator |
|  | Seal archiving boxes | Closure | Trial coordinator |
|  | Register all archived materials with named archivist | Closure | Trial coordinator |
|  | Monitor in preparation for secure disposal | Closure | Trial coordinator |
| **Abbreviations:** PMI, Project Management Institute; PPI, patient and/or public involvement; REC/HREC, research ethics committee; Aus, Australia; R&D, research and development; TGA, Therapeutic Goods Administration; MHRA, Medicines and Healthcare products Regulatory Agency; NHS, National Health Service; IMP, investigational medicinal product; GCP, good clinical practice; E, M & C, execution, monitoring and controlling; CRF, case report form.  *Specific to pharmacy trials. | | | |
